# Current landscape and future directions of synthetic biology in South America

**DOI:** 10.3389/fbioe.2023.1069628

**Published:** 2023-02-10

**Authors:** E. Sebastian Gomez-Hinostroza, Nicolás Gurdo, María Victoria Gracia Alvan Vargas, Pablo I. Nikel, María-Eugenia Guazzaroni, Linda P. Guaman, David J. Castillo Cornejo, Raúl Platero, Carlos Barba-Ostria

**Affiliations:** ^1^ Laboratorio de Investigación en Citogenética y Biomoléculas de Anfibios (LICBA), Centro de Investigación para la Salud en América Latina (CISeAL), Pontificia Universidad Católica del Ecuador, Quito, Ecuador; ^2^ The Novo Nordisk Foundation Center for Biosustainability, Technical University of Denmark, Kgs Lyngby, Denmark; ^3^ Department of Biology, FFCLRP, University of São Paulo, Ribeirão Preto, Brazil; ^4^ Centro de Investigación Biomédica (CENBIO), Facultad de Ciencias de la Salud Eugenio Espejo, Universidad UTE, Quito, Ecuador; ^5^ Glyxon Biolabs, Mexico City, Coyoacán, Mexico; ^6^ Laboratorio de Microbiología Ambiental, Departamento de Bioquímica y Genómica Microbianas, Instituto de Investigaciones Biológicas Clemente Estable, Montevideo, Uruguay; ^7^ Escuela de Medicina, Colegio de Ciencias de la Salud Quito, Universidad San Francisco de Quito USFQ, Quito, Ecuador; ^8^ Instituto de Microbiología, Universidad San Francisco de Quito USFQ, Quito, Ecuador

**Keywords:** SynBio, South America, DIY, Latin America, TECNOx, Open science

## Abstract

Synthetic biology (SynBio) is a rapidly advancing multidisciplinary field in which South American countries such as Chile, Argentina, and Brazil have made notable contributions and have established leadership positions in the region. In recent years, efforts have strengthened SynBio in the rest of the countries, and although progress is significant, growth has not matched that of the aforementioned countries. Initiatives such as iGEM and TECNOx have introduced students and researchers from various countries to the foundations of SynBio. Several factors have hindered progress in the field, including scarce funding from both public and private sources for synthetic biology projects, an underdeveloped biotech industry, and a lack of policies to promote bio-innovation. However, open science initiatives such as the DIY movement and OSHW have helped to alleviate some of these challenges. Similarly, the abundance of natural resources and biodiversity make South America an attractive location to invest in and develop SynBio projects.

## 1 Introduction

Synthetic biology (SynBio) is a multidisciplinary field that expanded enormously in the early 2000s by combining approaches, concepts, and tools of biology and engineering towards understanding, redesigning, and reprogramming biological systems in a controllable and predictable fashion, or even creating new-to-nature biological systems (Heinemann and Panke, 2006; Haseloff and Ajioka, 2009; Cameron et al., 2014). Among the pioneering studies within the domain of modern SynBio, two articles stand up, and they describe the construction of synthetic circuits from a similar set of parts (e.g., inducible promoter parts) for controlling the expression of the green fluorescent protein (GFP) gene in the model Gram-negative bacterium *Escherichia coli* (Elowitz and Leibler, 2000; Gardner et al., 2000). Since then, many other gene circuits, biological parts, model organisms, non-conventional cell factories, and approaches have been incorporated into the ever-growing SynBio agenda, mediating its transition towards a fully established (and still developing) discipline of scientific research and technological development.

Given that research in engineering and biological sciences has historically been led by universities and research centers in the United States, the United Kingdom, and other developed countries, it does not come as a surprise that the first SynBio-focused research groups emerged in those countries (Cameron et al., 2014). However, researchers from other regions, including South America, gradually joined the top SynBio laboratories worldwide (Bowen et al., 2016). After completing their training at different levels (either as Ph.D. students or postdoctoral researchers), some returned to establish their research groups in different South American countries, which marked the beginning of SynBio in the region (Nadra et al., 2020). In this regard, South America is a geographically vast and diverse region with countries known for staying at the forefront of discoveries and global scientific trends, e.g., Argentina, Chile, and Brazil. SynBio research groups had been established in these countries, mainly at the University of Buenos Aires, Pontificia Universidad Católica de Chile, and the University of São Paulo (Amores et al., 2015; Amarelle et al., 2019; Monteiro et al., 2020), respectively. Research teams from these institutions have published numerous articles developing new tools and methods for SynBio (Barone et al., 2017; Amarelle et al., 2019; de Siqueira et al., 2020).

Furthermore, Argentina, Brazil, and Chile have successfully established startup companies using SynBio tools. Alas, the reduced public investment in science, technology, and innovation and the highly bureaucratic and regulatory constraints of other countries in the region (e.g., Ecuador or Peru) have slowed scientific progress (Torres and Santos-Ordóñez, 2020).

Finally, South America offers a wide variety of natural resources that can be exploited through SynBio to push forward local bioeconomies^1^. The region has enormous potential for biomass production -extensive arable lands and suitable soils-which allows the development of several production chains across the countries. In the same direction, as part of the activities carried out in the primary sector (mainly agriculture and livestock), a large amount of waste biomass is generated that can be used as low-cost carbon sources to produce high-added value compounds^2^ (Vargas-García et al., 2021; OECD and FAO 2022). In the last 2 decades, a substantial increase in the market volume was is observed in the beforementioned strategic areas^3^ ^,^ ^4^. It is worth mentioning that areas such as biorefineries, agriculture, food and beverage production as well as active pharmaceutical ingredients (API) represent the major source of opportunities in the region (Niosi et al., 2013; Sasson and Malpica 2018; Keasling et al., 2021). Another aspect to take into account is the presence of a deep dichotomy between primary commodity-producing sectors and manufacturing industries. While the first is widely predominant, an expansion in technical and more advanced industrial capabilities are needed in order to capitalize the efforts into a greener bioeconomy (Mealy and Teytelboym 2022). SynBio can play an important role to strengthen the link between the primary sector and producing companies in the upcoming years.

## 2 Current landscape of SynBio in South America

### 2.1 iGEM, TECNOx, and other regional integration initiatives

**FIGURE 1:**
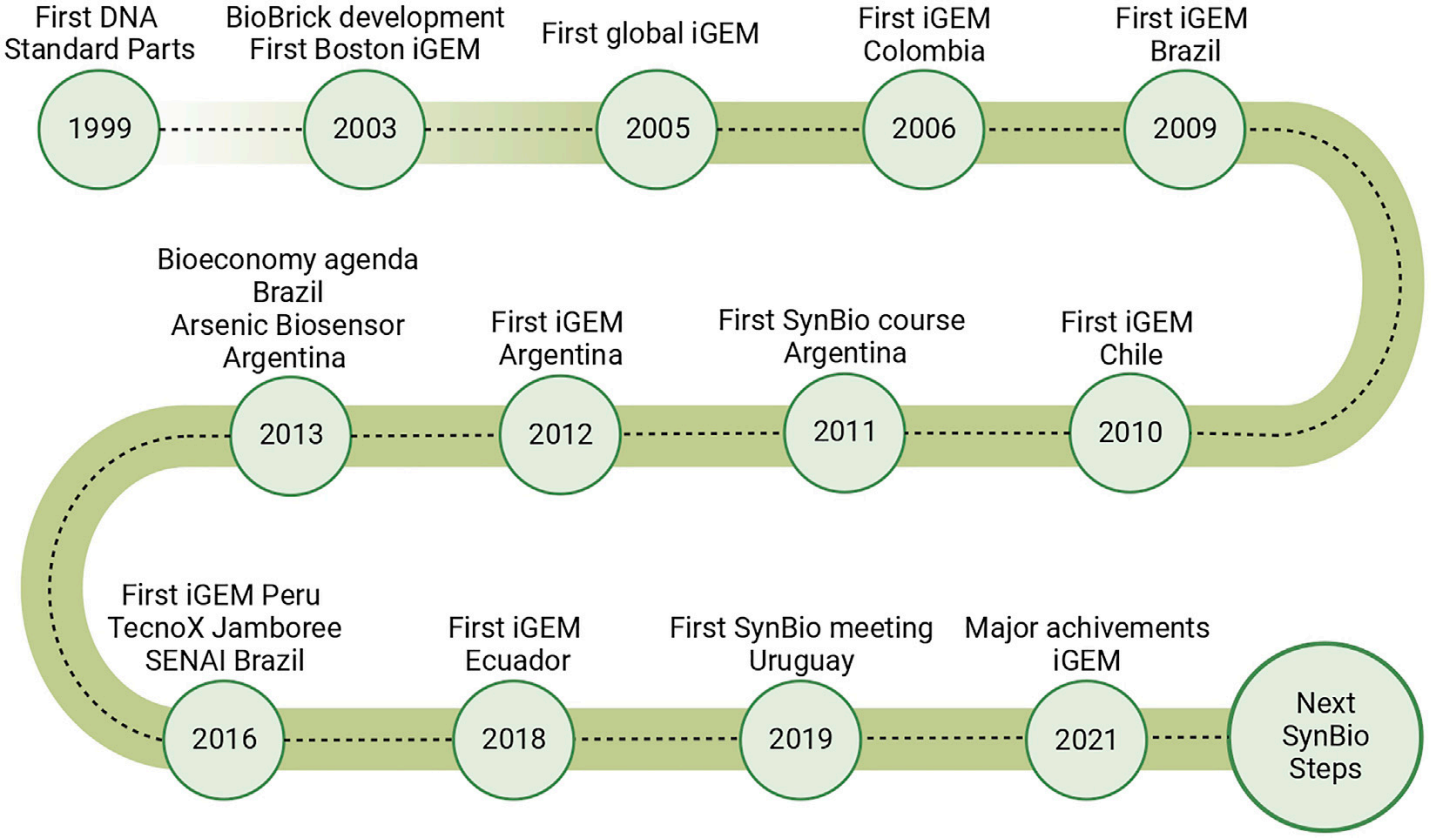
Collection of the leading milestones of SynBio globally and in South America.

Significant efforts have been devoted across the region toward boosting the development of SynBio activities. One of the most significant impacts of such endeavors, summarized in Figure 1, is the increasing level of participation of teams from South American countries in international competitions and events, e.g., the International Genetically Engineered Machine (iGEM), the iGEM design league, and TECNOx. The first-ever participation of a South American team in iGEM was registered in 2006 for a group of young researchers from Colombia^5^. Since then, teams from Argentina, Brazil, Colombia, Peru, Ecuador, Chile, Bolivia, and Venezuela have participated in iGEM^6^, or iGEM programs^7^. iGEM design league, is part of Leagues Program initiatives, enabling Latin American students to learn and apply SynBio by proposing solutions to local problems without needing a physical (and often costly) laboratory. This competition has created novel opportunities to expand SynBio in South America.

In the case of Brazil, the iGEM participation showed a significant increase over time. In 2009, a single team represented the country^8^ while in the 2019 edition (the last one based in Boston, United States, since travel restrictions were put in place due to the COVID-19 pandemic), Brazil was represented by six teams—a record number since the first Brazilian participation in the competence^9^. Most teams’ projects were directed to solve socially important and locally-relevant problems. Moreover, the teams are often organized in (and supported by) a very enthusiastic local network of researchers, i.e., the Brazilian Association of SynBio, aiming to enhance and spread SynBio information and to connect the private and public spheres. Additionally, Brazilian teams have earned six gold, four silver, and two bronze medals throughout their participation since2009^10^ ^,^ ^11^ ^,^ ^12^ ^,^ ^13^ ^,^ ^14^.

In Uruguay, efforts have been placed on bringing SynBio concepts to graduate and undergraduate students through the organization and participation in domestic and international courses. Some relevant examples are the Molecular and Cellular Bioengineering course, an undergraduate course dictated annually at the Faculty of Science, UdelaR, that includes a Synthetic Microbiology module^15^. Furthermore, the Synthetic Microbiology Symposium, organized in 2019, was the first postgraduate course entirely focused on SynBio in Uruguay^16^. This event was possible thanks to the active collaboration and joint efforts of researchers from the Environmental Microbiology Laboratory, Instituto de Investigaciones Biológicas Clemente Estable, Montevideo, Uruguay; the Systems and Synthetic Biology group, USP-Ribeirão Preto, Brazil; and the Environmental Synthetic Biology Laboratory, Centro Nacional de Biotecnología, Madrid, Spain. In addition to course organization, and reaching a wider audience, SynBio enthusiasts in Uruguay have been organizing plenary lectures and specialized sessions at national congresses since 2015. Examples of these events are the Pan American Society for Biochemistry and Molecular Biology 2015 Plenary lecture, given by Rafael Silva-Rocha from USP-Ribeirão Preto, Brazil, and the Synthetic Microbiology Symposium with the participation of Maria Eugenia Guazzaroni from USP-RIbeirão Preto, Brazil and Belén Calles form CNB, Spain, and the Opening Conference for the Uruguayan Society of Microbiology 2020 with a lecture by Pablo I. Nikel (an Argentinean researcher working as a SynBio group leader at the Technical University of Denmark).

SynBio in Argentina started to gain inertia at the beginning of the last decade. The driving force triggering this development has primarily been the dissemination of basic SynBio concepts in courses like “Introduction to synthetic biology”^17^. The first version of this pioneering course was held in 2012 at the Faculty of Exact and Natural Sciences (FCEyN), University of Buenos Aires. It has experienced a quantitative leap in the number of attendees ever since. The course focuses on the basics of the SynBio toolbox and its multiple applications. As a direct consequence of such learnings, several Argentinean teams have participated in iGEM competitions. In 2013, the Buenos Aires iGEM team built an arsenic biosensor using Biobricks available in iGEM kits (Barone et al., 2017; Amarelle et al., 2019; de Siqueira et al., 2020). Subsequently, several Argentinian groups were involved in the Latin-American iGEM version or TECNOx^18^ ^,^ ^19^ ^,^ ^20^. This event aims to address regional problems by applying SynBio tools. Even broader participation is expected in the next editions of the event thanks to the increasing interest of graduate students in this field.

Similarly, Chilean participation started in the 2010s with the 2012 UC Chile team and its project Luxilla Biolamp^21^. Later, there have been lectures about basic SynBio concepts by experts such as Ron Weiss at Adolfo Ibáñez University, to promote SynBio among young undergrad students^22^. Yet, one of the most important breakthroughs for SynBio development in the Andean country has been the adoption of formal courses about Synthetic Biology and its applications, for both undergrad and graduate students, at Pontificia Universidad Católica de Chile^23^ ^,^ ^24^.

Initiatives to welcome and develop SynBio in Peru have been scarce and are still young. Peru has participated in iGEM since 2016 with a team formed by high-school students^25^. Undergraduate students started participating in 2020 when long-distance attendance was again possible^26^. Theoretical knowledge of SynBio is also imparted in universities—although practical laboratory experience is virtually absent from most of these courses. Fortunately, the Peruvian government has promoted the dissemination of SynBio by organizing an international seminar in 2020; the scope of this seminar primarily focused on SynBio history, with a significant emphasis on its regulation and possible hazards to Peruvian biodiversity^27^.

Further North, in Ecuador, the situation has been quite similar. Interest in SynBio started with seminars about iGEM in2014^28^, with the subsequent formation of a SynBio undergraduate research team at ESPE University. Later on, in 2016, the first iGEM group was organized in Ecuador, and it took two extra years to participate in the iGEM Jamboree in Boston for the first time^29^. Nevertheless, these years of preparation paid off, as the team won a Silver Medal in their first attendance and a Gold Medal plus four awards in 2021 (more than any other team that year)^30^. Moreover, in 2022 the Ecuadorian Biotech EC team received six awards and a Gold Medal at the regional iGEM Design League^31^. In addition, SynBio courses have been taught at several Ecuadorian Universities, such as Universidad de las Américas (UDLA), Universidad de las Fuerzas Armadas ESPE, and Universidad Técnica Particular de Loja (UTPL); as well as at other government (Municipality of Quito and INSPI)^32^ ^,^ ^33^ and non-government organizations (CEBIO)^34^.

All the examples mentioned above are compiled in Figure 1, highlighting the importance of regional and international integration for the development of SynBio across South American countries. Figure 1 also demonstrates that countries with a long academic tradition (e.g., Colombia, Argentina, and Brazil) participated earlier at iGEM Jamborees. Additionally, as discussed in the next section, these countries have a higher impact on the SynBio setting in the region. In Table 1 the main awards and achievements of South American teams have been summarized.

**TABLE 1:** Awards and medals given to South American iGEM teams at Jamborees.

Country	iGEM team	Year	Award	Medal
Argentina	Buenos Aires	2013	Best Model	-
Bolivia	Bolivia	2021	Best Integrated Human Practices Inclusitiviy	Gold
Brazil	UFAM Brazil	2014	-	Gold
	USP-Brazil	2017	-	Gold
	Amazonas Brazil	2017	-	Silver
	Unesp Brazil	2018	Best Hardware	Gold
	USP-Brazil	2018	Best Measurement	Silver
	USP-Brazil	2019	-	Gold
	Sao Carlos-Brazil	2019	-	Silver
	USP Sao Carlos-Brazil	2019	-	Silver
	Amazonas Brazil	2019	-	Bronze
	UFRGS - Brazil	2019	-	Bronze
	USP - Brazil	2021	Best Plant Synthetic Biology	Gold
	Unila Latam - Brazil	2021	-	Gold
	UFMG_UFV_Brazil	2022	Best Hardware	Gold
Chile	UChile Biotech	2018	-	Bronze
Colombia	Colombia	2012	Regional Winner	-
	Colombia	2014	-	Gold
Ecuador^a^	Ecuador	2018	-	Silver
	Ecuador	2021	Best Food & Nutrition	Gold
			Best New Composite Part	
			Best Model	
			Best Sustainable Development Impact	

^a^ Team Ecuador 2021 had the best project in the Americas and reached the top 10 from more than 300 teams worldwide.

In the last decade, TecnoX, initially organized by former postdoctoral researchers Alejandro Nadra and Ignacio Sanchez at the University of Buenos Aires, Argentina, has been the seeding movement in South America that emulated the MIT iGEM competition but centered on local problems instead of replicating a top to bottom approach. TecnoX promotes the adoption of novel technologies to solve the region’s social problems (Nadra et al., 2020). Some of the projects achieved through past TecnoX editions include the development of a kit for detecting hemolytic uremic syndrome; creating a mapping app for avoiding energy shortages; an electronic sensor for detecting mercury in food and water; the design of a sensor for detecting melatonin deficiency; a low-cost sensor for the detection of dengue and hemorrhagic fever, among many others^35^. TecnoX is probably one of the most visionary initiatives in the continent to gather biotechnology students devoted to bringing forward immediate solutions to endemic challenges in the region. TecnoX is oriented to graduate students who will commit to developing a SynBio project for a few weeks (Nadra et al., 2020). Thus, participants outside academic organizations will find it challenging to engage in SynBio projects if they are not pursuing a degree.

### 2.2 SynBio in academic/research institutions

Building an economy centered on biotechnology, agriculture, and biodiversity (fueled by SynBio) will make it possible to replace products currently obtained from fossil sources with their bio-based counterparts (Head and Gray, 2016; Arun et al., 2021). Thus, searching, isolating, and developing new microorganisms (and the associated biochemical processes) to obtain products of interest is essential to establishing sustainable industrial processes. In this context, the concept of bioeconomy, which refers to the replacement of non-renewable raw materials with sustainable biological resources in industrial processes (Bugge et al., 2016; Töller et al., 2021), is increasingly relevant within the field. It has been a guiding factor for industrial activity not only for promoting sustainability but also for the enormous economic potential it presents: for example, it is estimated that by 2030 the bioeconomy will account for more than 7% of the market value in the United States (Guo and Song, 2019). Furthermore, according to data from the Organization for Economic Cooperation and Development (OECD), the bioeconomy mobilizes immense capital flows (around 2 trillion Euros) on the world market and generates around 22 million jobs, with an estimated creation of 1 million new jobs by 2030 in Europe^36^. Thus, it is not surprising that governments and industrial entities have been promoting technological development in emerging disciplines, such as SynBio, to consolidate the bioeconomy (Flores Bueso and Tangney, 2017; Wei et al., 2022).

Following this trend, in 2013, the national confederation of Brazilian industry (CNI) released the report “Bioeconomy: an agenda for Brazil”, in which an analysis of the bioeconomy in the Brazilian scenario was carried out in order to identify obstacles and opportunities for the increase in national competitiveness^37^. Hence, the development of the bioeconomy represents a promising opportunity for Brazil (extensible to Latin America), as a country with a large arable area, developed agriculture, a successful experience in biofuels, and high availability of biodiversity^38^. Estimated that the bioeconomy represented US$286 billion in 2016—approximately 14% of Brazil’s GDP.

Considering the foregoing and recognizing that combining SynBio approaches with the enormous Brazilian natural wealth would enhance the arising of new bioproducts, and funding agencies and research institutions provided essential resources aiming to encourage SynBio projects in academia and business^39^. Thus, although efforts were directed to consolidate investments in infrastructure (such as the SENAI Innovation Institute for Biosynthetic) and qualification of human resources, there is still a limitation in the latter. Most of the scientific community is still limited to basic projects, nucleated in iGEM projects with the active participation of predominantly undergraduate students. Although some companies (mainly startups) in Brazil have been working on projects related to SynBio, they have not yet validated their potential in the market or assembled world-class SynBio teams.

Still, a few research groups produce relevant SynBio studies that significantly impact the international scientific community. Brazil’s primary SynBio research node is hosted at the University of São Paulo (USP) at Ribeirão Preto. The origin of this hub is related to the completion of Rafael Silva-Rocha’s doctorate in Victor de Lorenzo’s group (Madrid, Spain), a European pioneer group. For example, Silva-Rocha’s group (FMRP-USP) has established computational tools based on genetic algorithms for the design of complex synthetic promoters in bacteria that display new-to-nature regulatory control at the promoter (Guazzaroni and Silva-Rocha, 2014; Amores et al., 2015) or protein level (Monteiro et al., 2020). These works, and others characterizing complex promoters, have revealed a lack of information on the process of combinatorial regulation at the promoter level in bacteria (Monteiro et al., 2018; Monteiro et al., 2020; Sanches-Medeiros et al., 2018).

Similarly, Guazzaroni’s group at the FFCLRP-USP has been developing novel experimental approaches for mining building blocks by applying concepts in the interface of SynBio and metagenomics (Westmann et al., 2018; Amarelle et al., 2019; Nora et al., 2022). Also, SynBio approaches were used to engineer enhanced acidic resistance in *Escherichia coli* by characterizing a collection of unique synthetic circuits (de Siqueira et al., 2020). The latter study was emphasized in the “Research Highlights–Synthetic Biology” section of Nature Chemical Biology (Deane, 2020). It is worth noting that both groups (Silva-Rocha and Guazzaroni) jointly organized the I, II, and III Workshop on Systems Microbiology and Synthetic Biology, international events held at USP in 2015, 2017, and 2019, with the participation of renowned national and international speakers (i.e., researchers from the University of Edinburgh-Scotland, Instituto Clemente Estable from Montevideo-Uruguay and the University of Cambridge-England).

Argentina is on its way to consolidate a central pillar: the bioeconomy^40^. In alignment with Brazil, Argentina is focusing on establishing a strong foundation in the bioeconomy. In order to bolster this scientific framework, Argentina allocates 0.2%–0.4% of its GDP, with 80% of these funds allocated to the fields of Engineering, Agriculture, Material Science, Biology, Health, and Natural Science^41^. These areas are crucial in the advancement of disciplines such as SynBio. Currently, Argentina is in the preparatory stage of further developing SynBio, as there is significant potential for the exploitation of SynBio projects within the country.

In the last 20 years in Argentina, significant advances have been achieved in sustainable development, research, and innovation–including several niches across the different productive areas (mostly biotechnology, agriculture, and biomaterials)^42^. Since then, many initiatives have focused on promoting knowledge-based areas and generating added value in the extensive productive supply chain. This policy is being implemented, and it is aligned with a techno-productive development model towards2030^43^. Another critical aspect and relevant fact pushing this transition forward include the collaboration between public and private sector entities in many multidisciplinary projects. This continuous interaction created an innovation hub of Argentinian startups and strengthened the existing enterprises and institutions that paved the way to a greener society^44^.

SynBio research in Argentinian academic institutions is slowly gaining momentum with the support of research groups, such as the one led by Alejandro Colman-Lerner, Alejandro Nadra at the University of Buenos Aires; Hugo Gramajo, Cristian Suárez as well as Larisa Estefanía Cybulski at the Institute of Cellular and Molecular biology of Rosario-IBR (National University of Rosario). Colman-Lerner’s group explores gene expression systems (Givré et al., 2022), while Nadra’s group focuses on developing biosensors (Barone et al., 2017), and Gramajo’s group in exploring the potential of novel organisms in polyketide and fatty acids biosynthesis (Roulet et al., 2018; Livieri et al., 2019). Currently, the country is following a learning process and becoming familiar with the SynBio tools that are available in the field.

In the last decade, Chile has invested significant public and private resources in science and technology, enabling significant scientific development in the region. During these years, investment in R&D has been stable at a 0.3% of GDP^45^. An important source of investment is the government-led initiative Fondo de Financiamiento de Centros de Investigación en Áreas Prioritarias (FONDAP), which was created in 1997 and has provided dozens of millions of dollars yearly ever since^46^ ^,^ ^47^. Additionally, the Ministry for Planning and Political Economy supported the creation and operation of the Millennium Nucleus Center for Plant Functional Genomics, a lab that specializes in plant systems and synthetic biology^48^. This investment translated into breakthroughs in, among other areas, SynBio. A Synthetic Biology group at Pontificia Universidad Católica de Chile, led by Fernán Federici, has developed or been involved in novel progress in SynBio, such as DNA assembly tools (Pollak et al., 2020), Cell-Free expression and RNA sensing systems (Arce et al., 2021; Guzman-Chavez et al., 2022), genetic tools for marine protists (Faktorová et al., 2020), improving soil microorganisms for agricultural applications (Zúñiga et al., 2018), and data analysis tools (Yáñez Feliú et al., 2021).

On the other hand, in 1984, Uruguay created the Programa de Desarrollo de Ciencias Básicas (PEDECIBA) to promote the research and education in biology, physics, mathematics, chemistry and geosciences, and basic sciences^49^. This program together with the creation of the Faculty of Science and other more recent public efforts, such as the National Agency for Investigation and Innovation (ANII), allowed the consolidation of research groups around molecular and structural biology, imageneology, proteomics, computational biology, among others, that represent the basis for the development of SynBio^50^. More than half of the first-rate scientific research is produced at the Universidad de la República, while the rest is mainly produced at research centers like the Instituto de Investigaciones Biológicas Clemente Estable (IIBCE), Instituto Nacional de Investigaciones Agropecuarias (INIA) and Institut Pasteur de Montevideo (IPMOnt)^51^. In these institutes, several research groups are dedicated to microbiology, structural biology, molecular biology, biochemistry, molecular modeling, bioinformatics, microscopy, and protein engineering, preparing a fertile field for developing SynBio.

Nevertheless, there is still a reduced and dispersed number of researchers focused on this field. Examples are found in the Faculty of Science where Gonzalo Moratorio explores the use of SynBio to study the experimental evolution of viruses (Moratorio 2020; Pintó et al., 2021), Vanessa Amarelle from IIBCE that of seeks new “regulatory parts” from metagenomic libraries in collaboration with Guazzaroni group at USP Ribeirao Preto (Westmann et al., 2018; Amarelle et al., 2019) and Raúl Platero that participate in the assembly of modular vectors with standard architecture during its postdoc in Victor’s de Lorenzo group in Madrid (Silva-Rocha et al., 2013). A close inspection of funded projects by the Uruguayan Agency for Investigation and Innovation allows us to identify other groups strongly working in SynBio approaches, such as Felipe Trajtemberg groups^52^ that exploit its experience in structural biology to design new sensing/response devices (Lima et al., 2021). Uruguay has seen a considerable investment of state resources in education (≈4.5% of the gross domestic product, GDP), as well as in research and development (≈0.5% of the GDP)^53^.

SynBio is making significant contributions to the industry in many world countries. However, Uruguay is far from implementing SynBio approaches in the industry, partly due to the lack of two pillars for bioinnovation: a solid local high-tech industry and a critical mass of researchers dedicated to SynBio among the local scientific community. Based on the comments from Section 2.2, it is expected that in the coming years, a new generation of researchers will establish the basis of a SynBio-based industry in Uruguay.

Similarly, Ecuador has invested resources in developing microbiology, molecular biology, and SynBio technologies. Mainly since 2003, when the Ecuadorian government created the Ecuadorian Corporation for the Development of Research and Academia (CEDIA in Spanish)^54^. This institution has sponsored scientific projects through direct funding each year. This effort has, nevertheless, been focused mainly on its agricultural sector and conservation (Torres and Santos-Ordóñez, 2020). As well as in other countries with access to the Amazon rainforest, genetic resources from native Ecuadorian species have shown potential for therapeutic and industrial applications (Morocho et al., 2018; Proaño-Bolaños et al., 2019; Sánchez García and Quilumbango Grijalva, 2021). Thus, Ecuadorian genetic diversity is a promising source for bioparts with novel characteristics for synthetic biology.

SynBio research in Ecuador has followed a similar path to Uruguay, with a few institutions conducting SynBio-related research. The Centro de Investigación Biomédica at Universidad UTE (CENBIO-UTE) and the Universidad de las Fuerzas Armadas ESPE (ESPE) are at the forefront of SynBio in Ecuador. For example, in collaboration with researchers of the Instituto de Ciências Biomédicas in USP, researchers at CENBIO, developed novel tools for engineering the non-model Gram-negative bacterium *Burkholderia sacchari* (Guamán et al., 2018). ESPE has been a critical node for iGEM teams and agrobiotechnology applications. On the educational side, the creation of a master’s degree in Synthetic Biology opens up new opportunities for the training of researchers in the field. Plus, interest in biotech is ever-growing thanks to several universities offering Biotech Engineering or related careers to local students^55^ ^,^ ^56^ ^,^ ^57^ ^,^ ^58^ ^,^ ^59^ ^,^ ^60^.

### 2.3 SynBio in industry

The development of SynBio in the region is still in its infancy, as there are no consolidated companies that apply this technology to their R&D^61^. Nevertheless, during the last decade, many startups were created and started to play a pivotal role in the development of industrial SynBio. In the case of Argentina, the Argentine Chamber of Biotechnology (CAB) gathered 67 startups that pursue integrating new biotechnology-based companies with the support and networking of the largest companies in the biotechnology sector^62^. Among these startups, Inmet - “Ingeniería metabólica” represents a clear example of a biotechnology-based startup that integrates a SynBio platform to design, build and optimize cell factories for multiple purposes. In particular, Inmet uses several SynBio tools and approaches (synthetic promoters, targeted multiplex genome editing systems, precursor supply optimization, and feedback/forward regulation control), and they have established a systematic and iterative workflow that allows an efficient product development process. In addition, Inmet collaborates with key players in biotech to develop and produce high-value compounds such as polyhydroxyalkanoates (PHAs), biodiesel, natural preservatives, biopesticides, and enzymes for the food industry among others^63^.

In 2019, Syocin was founded as a successful venture-backed startup in California with subsidiary businesses in Argentina. The company specializes in producing protein-based bio-bactericides using a platform that combines *in silico*, *in vitro*, and *in vivo* methods to target various bacteria. This platform also utilized SynBio tools, allowing for versatility in designing the desired biomolecule^64^. Andes, another startup founded in Chile and now based in California, developed a system for introducing symbiotic bacteria into plant seeds to increase yield^65^. The company plans to incorporate SynBio into this system to engineer the bacteria and improve host-microbe interactions^66^. In Ecuador, SilicoChem, a startup spun out of UTPL, focuses on producing omega-3 fatty acids through genetically modified *Saccharomyces cerevisiae* ^67^.

In South America, biotech accelerators such as Ganesha Lab in Chile and GridX in Argentina have provided support and funding to emerging biotech companies^68^ ^,^ ^69^. However, these accelerators do not offer access to research facilities or specialized business training like those found in developed nations, such as IndieBio, StartCodon, Startup + Health, and 37 Angels. The biotech sector in South America still lacks mature venture capital options that can address the diverse needs of biotech startups, including consulting services for regulatory and intellectual property issues and access to equipped technical facilities^70^. Despite this, these limitations present opportunities for potential investors in the region.

The impact of SynBio on the South American economy has so far been limited, with no major projects utilizing the region’s strengths, such as its commodity market. However, this trend could change if iGEM ideas are developed into market-ready products or services. A list of SynBio startups can be found in Table 2.

**TABLE 2:** Examples of South American startups that rely on SynBio.

Startup	Area	Country	URL
Andes	Agrotech	USA/Chile	https://www.andes.bio/platform/
Inmet	Optimization of cell factories	Argentina	https://inmet.com.ar/
Syocin	Crop protection	USA/Argentina	https://syocin.com/
SilicoChem	Fatty acid metabolism	Ecuador	https://www.silico-chem.com/

### 2.4 Citizen and open science

SynBio tools and innovations have impacted art, education, environmental monitoring, and the fashion industry through the D-I-Y (Do it Yourself) movement (Landrain et al., 2013). Members or “biohackers” are usually amateur biologists aiming to develop easy-to-use and affordable techniques or products or teach biology and molecular biology outside academia (Seyfried et al., 2014). Because of this, more and more scientists and people with less or no formal education have learned and applied SynBio, usually in community labs (Bennett et al., 2009; Delgado, 2013). Nevertheless, assembling a DIY laboratory with little to no outside funding represents a significant challenge. Thus, equipment from FabLabs and Open-Source Hardware has filled the void caused by the unaffordability of traditional instruments^71^.

#### 2.4.1 Open-source hardware and scientific equipment in Latin America

In contrast to highly industrialized economies, Latin American nations chronically lack access to a local surplus of laboratory infrastructure, relying heavily on foreign manufacturers and importing most of their equipment and supplies. Local industries possess limited technical expertise and less incentive to mass manufacture full-length versions of advanced scientific instrumentation^72^.

In many Latin American countries, the prolonged importation, logistic processing, and associated costs of mass-manufactured equipment impact its availability and practical operability (Thomaz and Mormul, 2014). In addition, further training and post-sales services are often required to start or maintain its operation, delaying the immediate implementation of those technologies. For those reasons, Open-Source Hardware (OSHW) devices could become fundamental allies for accessing scientific instrumentation with more advanced technological features as an alternative to traditional acquisition channels (Nuñez et al., 2017; Bravo-Martinez, 2018). In contrast, patented technologies and devices are usually regarded as “black magic boxes” with sparse personnel adequately trained to operate them and fewer commercial options. This situation poses substantial limitations not solely in their costly operations and imposes a barrier to genuinely gaining widespread technical dexterity and understanding of the technology behind advanced scientific instruments.

OSH does not just represent an economical option that offers further customization, and a rapid distribution process with flexible intellectual property attributes. It also allows additional project integration due to its modularity (Gavras and Kostakis, 2021). Furthermore, the DIY construction process enables its users to experience the technical details by finding training through the assembly process (Ravindran, 2020).

It is common to find commercially available microcontrollers and components enlisted in most of the bill of materials of many OSHW projects: Arduino, RaspberryPi, Adafruit, *etc.* ^73^. Nevertheless, in an economically diverse and complex region like Latin America, the availability of imported components and consumer electronics mentioned above varies among countries. While more developed nations have large domestic markets for imported microcontrollers (e.g. Chile, Brazil, and Uruguay), in some other countries (Peru, Paraguay, and Argentina), these components could become prohibitively expensive due to complex regulatory requirements, inadequate logistic infrastructure, and chain supply disruption that increase pressure in their trade balances. These scenarios generate demanding taxation and uneven regional pricing^74^ ^,^ ^75^ ^,^ ^76^ ^,^ ^77^ ^,^ ^78^ ^,^ ^79^. Instead, local initiatives have emerged even in states severely affected by unfavorable economies, such as Venezuelan “Pingüino-VE”^80^. In recent years, local entrepreneurs have emerged to provide locally-assembled equipment and supplies but still have reached a limited market share in the region. In the case of additive manufacturing (3D printing), the most encouraging developments have been led by Brazil (Metamaquina)^81^. Both countries have embraced entrepreneurship by developing and manufacturing low-cost machines, software, and supplies. Metamaquina is oriented toward an open-source hardware business model, allocating most of the assembly instructions to Open-source repositories.

In Latin America, Brazil, Chile, Mexico, and Argentina have a prominent role in generating the largest output of scientific production related to additive manufacturing technologies. Still, it only has 2% of global scientific production (Rodríguez-Salvador et al., 2019). Regarding bioprinting technologies focused on future 3D tissue culture and organ printing, Brazil leads the number of scientific publications (Rodríguez-Salvador et al., 2019).

#### 2.4.2 SynBio, DIYBio initiatives, and open hardware

Outside of the academic context, the open hardware movement and SynBio in Latin America have a very heterogeneous development that addresses different needs and scopes and, in many cases, cannot be entirely separated from academic organizations^82^. Two significant interests could be centered on developing biotechnological tools: 1) Open-source hardware and 2) SynBio components^83^ ^,^ ^84^ ^,^ ^85^ ^,^ ^86^ ^,^ ^87^ ^,^ ^88^ (Federici et al., 2013; Lewens, 2015). One of these emergencies is constructing scientific equipment in a region that is chronically scarce or difficult to obtain. Due to high costs and a stricter regulatory framework that limits access to genetic modification technologies and suppliers (Kuiken and Kuzma, 2021), SynBio has had limited practical penetration in independent laboratories or maker spaces. Therefore, many existing developments focus on education using readily available biomaterials and supporting entrepreneurship^89^ ^,^ ^90^ (Valenzuela-Zubiaur et al., 2021).

There are hybrid initiatives organized by academic consortiums that have extracurricular activities that promote the value of open science: This is the case of “Laboratorio de Tecnología Libre” or Free Technology Lab^91^ and Red Fungi^92^, a network for several labs belonging to the University of Chile exploring biomaterials, and essentially promoting trans-disciplinary studies between engineering, material science, conservation, and design within a ludic and informal environment. Likewise, the University of Chile possesses accelerators and innovation labs that offer most of the digital manufacturing tooling to promote entrepreneurship and Open Science developments^93^.

Civic organizations thriving in non-academic contexts are usually Fab Labs oriented to education and the training of tooling and equipment in areas where its operation still represents a limiting factor. An extensive network of Fab Labs across Latin America awaits major visibility and awareness^94^. The largest concentration in South America is allocated in the San Paulo metropolitan area, Brazil, followed by Chile and Colombia^95^. A particular mention of Fab Labs that have dedicated programs for teaching synthetic biology and the assembly of scientific equipment is the UTEC Bioacademy and Open BioFab Lab Lima in Peru^96^ ^,^ ^97^. After the HTGAA (How to Grow Almost Anything) conferences in 2017 organized by the MIT Media Lab and David Kong’s Community biotechnology lab, the latter is the resulting effort. The programs aim to teach science in vulnerable communities and build low-cost scientific instruments.

#### 2.4.3 DIYBio, amending the gender gap

Other associations are oriented toward networking and pushing gender equality and women’s participation in science; this is the case of Biotech Latina with a dynamic organization by Sofia Taday^98^. One of the network’s objectives is to facilitate and promote the access and work of early-career female scientists that have been historically neglected in scientific endeavors (Wenneras and Wold, 1997).

It is important to address the outstanding participation of young women as scientific entrepreneurs engaging in providing services and products resulting from the immediate applicability of biomaterials. Few examples are Labva Laboratorio de Biomateriales de Valdivia Chile led by Maria José Besoain^99^ and Etimo Biomaterials in Argentina coordinated by Camila Castro^100^. Colombian artist Heidi Jalk, currently leading the Sistemas Materiales research group at the University of Buenos Aires, has focused her work and research on bio-inspired, sustainable materials^101^. The intersectional research and entrepreneurship of these young female leaders could help to redefine aspirational role models in a region still struggling with enormous gender inequality and low representation of women in science. The social impact of more female representation in leading scientific and creative positions in South America could catalyze a transformative benefit for increasing regional human development indicators^102^.

A comprehensive compilation of groups and their contributions to Open and Citizen Science has been summarized in Table 3.

**TABLE 3:** Citizen and open science in South America. There are several groups, mainly in Argentina and Chile, that encourage the development of SynBio in society.

Group	Main contributions	Country	URL
Cadastro Maker Fazedores	Interconnection for small businesses	Brazil	https://blog.fazedores.com/cadastro-maker/
Metamáquina	Open-source hardware development	Brazil	https://github.com/Metamaquina
Laboratorio de Tecnología Libre	Open science promotion	Chile	https://federicilab.org/about/
Red Fungi	Biomaterials development	Chile	https://redfungiblog.wordpress.com/que_hacemos/
U Chile accelerators	Open science development	Chile	https://centrodeinnovacion.uc.cl/la-reactivacion-de-la-red-chilena-de-fablabs/
Biotech Latina	Gender equality in SynBio	Ecuador	https://www.facebook.com/BiotecLatina/
Labva	Biomaterials development	Chile	https://www.labva.org/somoslabva/
	Gender equality		
Etimo	Biomaterials development	Argentina	https://etimobiomateria.wixsite.com/etimo
	Gender equality		
UBA Sistemas Materiales	Biomaterials development	Argentina	https://www.biodesignchallenge.org/uba-2021
	Gender equality		

## 3 Development of other elements of a bio-innovation ecosystem in the region

An important aspect that supports the advancement in SynBio in obtaining and understanding the role of new genetic tools and parts is the application of Next-Generation Sequencing (NGS) (Hughes and Ellington 2017; Bashor and Collins 2018). By analyzing massive amounts of data obtained from libraries of organisms with particularly useful properties, novel circuits can be engineered. For that reason, the increase in NGS use in South America may positively impact research in SynBio.

The Latin American NGS market is expected to grow at a compound annual rate between 6% and 16% over the next decade, reaching a net valuation of about $715 USD million by the early 2030s^103^. The speed, throughput, and precision of NGS have revolutionized genetic analysis, allowing it to be used in new fields like genomics and clinical research, reproductive health, environmental, agricultural, and forensic science. This ramp-up in the investment in nucleic acid sequencing technology echoes those seen in the early years of the beginning of the Human Genome Project more than 30 years ago (Wiechers et al., 2013). Although, this time, the market is focused on personal genomics and industrial applications^104^. The Latin American NGS business is rapidly expanding as genome mapping efforts intensify. A few examples are discussed in the following paragraphs.

In 2020, 1,433,519 new cancer cases were reported in the region, according to the Global Cancer Observatory (GLOBOCAN) and the National Center for Biotechnology Information (NCBI)^105^. Furthermore, by 2040, these figures are predicted to rise by 78%, to more than 2.5 million people afflicted with cancer each year (Alvarez-Gomez et al., 2021). According to GLOBOCAN studies, the cancer burden in Latin America and the Caribbean is anticipated to rise among those 65 and older. As a result of the rising prevalence of genetic illnesses in the region, governments are undertaking genome mapping programs, fueling the demand for sequencing products.

The study about the genomes of 280 Native Americans from Peru was published in June 2018 (Harris et al., 2018) as part of the Peruvian Genome Project by a team from the United States, Peru, and Brazil. The purpose of this research was to examine and comprehend Peruvian demographic trends and their historical background. The impacts of this study have helped to obtain a more detailed picture of the Peruvian population’s epidemiology (Carrillo-Larco et al., 2022) and add further focus on pediatric genetics, cancer genetics, autoimmune diseases, and neurogenetics. This could facilitate the adoption of more refined theragnostic applications (Harris et al., 2018).

“DNA do Brasil” was initiated in December 2019 with the goal of sequencing the DNA of 15,000 Brazilians aged 35–74. DASA, a Brazilian medical diagnostics firm, has absorbed the costs of the sequencing for the first 3,000 samples, while Illumina, will supply the genome analysis inputs and Google Cloud will store the data^106^. The project's main goal is to use genetic analysis to treat and prevent genetic illnesses in the country.

Another important niche of expansion of the NGS market in Latin America is expected to increase as more drug discovery platforms use NGS technology in precision medicine and genomics. Various projects connected to drug development, research, and clinical diagnostics procedures are continuing in the region due to NGS capabilities, rising applications, and the developing trend of personalized medicine. As of 2017, 221 NG platforms and 118 research groups in Latin America were working on cancer genomics projects (Torre et al., 2017; Oliver et al., 2019; Herzog et al., 2021).

Various projects have been launched in Brazil to study HIV genetic diversity through the generation of HIV complete or near full-length genomes (NFLG) and improve the characterization of intra- and interhost diversity of viral populations, helping to assess the antiviral drug resistance in geographically defined populations (Alves et al., 2019). Similarly, Porcine circovirus type 3 was detected in Chile using Next-Generation Sequencing (NGS) (Ariyama et al., 2021). This simultaneous identification of pig viral species utilizing its RNA and DNA genomes can be valuable for detecting swine viral emerging illnesses.

Similarly, NGS is utilized in Colombia to identify, diagnose, and control viral illnesses in plants in order to develop plants that are resistant to viral infections. This later effort is the result of hybrid interaction between the academic and private sectors. The EAFIT through the Axomics Sequencing Center, a spin-off provides industry solutions based on genomic data analysis for agricultural, and biotechnological research. The center has provided important information regarding the strain diversity of *Helicobacter* pylori-associated with chronic gastritis (Muñoz et al., 2021) and the determination of cacao fungi infection (Guerra Sierra et al., 2022).

## 4 Opportunities and future perspectives of SynBio in SA

From the examples and activities discussed above, it becomes clear that South American countries have already started a regional SynBio revolution. Brazilian and Argentinian governments are providing the essential funding to keep local research groups and businesses afloat^107^. This effort translates into more publications and startup companies than elsewhere in the subcontinent^108^. Moreover, cooperation and integration among research groups from different countries may overcome local regulatory constraints or the relative lack of funding. In the future, these occurrences may help develop the necessary capabilities to conduct SynBio research more evenly and to a higher level of technical complexity.

Furthermore, with its contrasting climates and vast areas suited for agriculture, South America is a major producer and exporter of food and feed (OECD and FAO, 2019). Sugarcane is a prime example of an important crop for local economies (mainly in Brazil), and it constitutes an almost ideal raw material for microbial fermentations (de Araujo Guilherme et al., 2019; Pratto et al., 2020). If used locally, the cost of using these raw materials for growing biomass or fermenting high-value chemicals is low, thereby positively impacting bioproduction.

Yet, the dominant perception of the large majority of Latin American governments is not positive on embracing genetic modification technologies and Synbio products into their agroforest supply chains Sharry and Briones 2020). However, that political positioning is contradictory to the observed trend that incorporates sub-products and derivatives of GMOs into ingredients and components of further markets including food additives, medicines, and fibers (Turnbull et al., 2021). Brazil and Argentina are two of the top five producers of GM crops in Latin America, according to the “International Services for the Acquisition of Agri-Biotech Applications” (ISAAA)^109^. In the 2019 report, Paraguay, Colombia, Chile, Bolivia, Argentina, and Brazil collectively accounted for 44% of the world’s GM cropland, which is comparable to North America’s (44% in the US and Canada) share. It is important to highlight that Brazil alone produced more GM soybeans than the US did. Chile is currently the biggest exporter of transgenic seeds in the southern hemisphere, while Colombia dramatically expanded the cultivation of transgenic maize and cotton by around 15%.

Additionally, excluding the United States and Mexico, South America contains five of the seven megadiverse countries of the Americas^110^. Nevertheless, the platforms involved in assessing and exploiting the regional biodiversity greatly divert in their implementation and even their ideological and political liaisons. Still, bioprospection of novel chemicals for pharma and industry may provide South America with new opportunities to develop its secondary sector on the groundwork of SynBio and Biotech. Bioprospection is a practice that seeks to explore the immediate commercial value of the biodiversity of a less economically developed country with the assistance of more technologically advanced countries and their biotechnological industries (Torres and Velho 2009). This practice is not always rewarded as advantageous or fair for developing nations but (according to Torres and Velho, 2009) has been demonstrated to be in some cases a successful pathway for economic development, like in the case of Colombia.

Nearly 120 bioactive molecules from Colombian forest plants have been identified and some have been approved for further patent application and commercialization (Torres and Velho 2009). The same report, however, suggests that even mega biodiverse countries like Colombia have become been greatly benefitted from their interaction with large pharmaceutical companies, and academic groups through the acquisition of equipment, and training personnel, the radical failure can be addressed in the minimal participation of local business participation and return benefits for the indigenous communities that withholds the traditional knowledge. This is nevertheless, just one example of opportunities for bioprospection in the region, as there are other novel chemicals that may be exploited commercially (Vasconcelos et al., 2018; Delgado-García et al., 2019; Proaño-Bolaños et al., 2019).

On the other hand, there are structural challenges to tackle urgently regarding the regulatory, financial, educational, and economic frameworks that could be reverted to ease the current conditions of the region and could have an immediate and long-lasting impact (Torres and Santos-Ordóñez 2020). Among those conditions are the protectionism measures some local markets impose on the importation of equipment, reagents, and materials needed for even the most basic operations for research (Nhamo et al., 2021). Another one is the educational landmarks that need to be achieved in order to give access to STEM students in Latin America adequate resources for an education of quality (Sahin and Mohr-Schroeder 2019).

**FIGURE 2:**
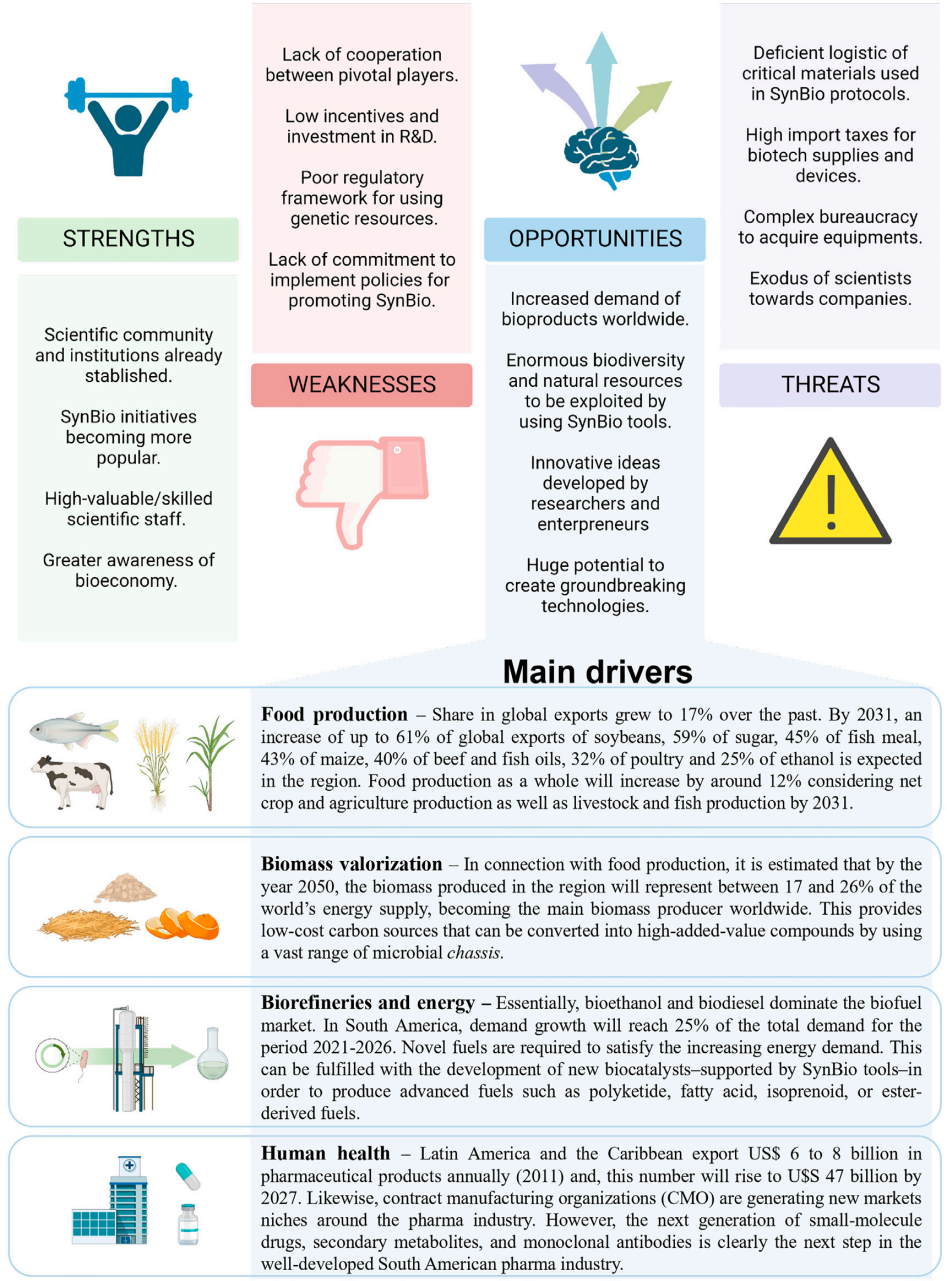
Main strengths, weaknesses, opportunities, and threats of SynBio in South America.

Ultimately, the development of Open-source Technologies and Free Hardware might break the dependence on expensive foreign equipment, which has dragged several regional developments. Local machinery has the potential to replace unavailable instruments, the absence of which stalls development or reduces the quality of research. Thus, by decentralizing production, the technological gap between South American SynBio and other parts of the globe could be bridged. Likewise, research groups from this subcontinent could benefit from local biodiversity to obtain bioparts and biological chassis, paired with the need for governments to replace commodities as the main source of revenue^111^,^112^ (Ocampo, 2017), and the (double-edged sword) of globalization and supply chains. A summary of the opportunities and potential threats for the development of SynBio in South America is illustrated in Figure 2.

## 5 Concluding remarks

The mature field that we know as SynBio consolidated at the onset of the 21st century, and the South American counterpart to these developments emerged in the region in the 2010s (Figure 1). This delay was further worsened by the lack of solid scientific funding in some countries, which, in some cases, led to serious financial difficulties for researchers working in Ecuador and Peru. Nevertheless, all South American countries invest approximately the same GDP in research (around 0.5%). This number might be misleading, as the sizes of the economies are vastly different (i.e., the GDP of Brazil is 1.4 trillion USD for Brazil and 53 billion for Uruguay)^112^. Thus, the total amount of money allocated to research will widely vary depending on the country. Despite these economic issues, Brazil, Chile, and Argentina have paved the way for developing SynBio in the region.

Advances in the design of synthetic promoters and other functional parts, the mining of building blocks for SynBio, the development of *E. coli* strains endowed with high levels of acid resistance, microbial biosensors, and the biotechnological implementation of novel bacterial platforms are just but a few examples that bear witness of the reach of SynBio. More projects could be done with a higher level of integration and cooperation between institutions from different countries. Furthermore, the expansion of Open Hardware in the region, the establishment of instrument manufacturing companies, and the creation of FabLabs in Brazil, Colombia, Chile, and Peru have all reduced the reliance on costly foreign equipment. Furthermore, initiatives such as iGEM and TecnoX will continue to promote the interest of younger generations of researchers that represent the future of SynBio and which in turn, creates novel ideas for solving regional problems. Finally, in addition to the initiatives that have already been mentioned, it is key to strengthen SynBio by promoting the participation of researchers of different countries in research projects, aimed at proposing solutions to regional problems, based on the application of strategies of synthetic biology, agrobiotechnology, as well as other scientific disciplines.

## Footnotes

1 Levanchuk, M. (2020). Bioeconomy in Latin America and the Caribbean concepts, international and regional cooperation, prospects for business. Sociedade Portuguesa de Inovação.

2 Rodríguez, A. G., Rodrigues, M., and Sotomayor, O. (2019). Towards a sustainable bioeconomy in Latin America and the Caribbean. Santiago de Chile: United Nations.

3 Cálix, Á., and Blanco, M. (2020). Los desafíos de la transformación productiva en América Latina. First., ed. E. Martínez México: Friedrich-Ebert-Stiftung.

4 Vargas, V., Rama, M., and Singh, R. (2022). Pharmaceuticals in Latin America and the Caribbean Players, access, and innovation across diverse models. Washington, DC: World Bank.

5 iGEM Foundation (2006). 1st Synthetic Biology and iGEM South American Team 2006. South American Team 2006. Available at: https://2006.igem.org/wiki/index.php/Latin_America_2006 [Accessed 29 August 2022].

6 iGEM Foundation (2022). Team List For All Years. Team List For All Years. Available at: https://old.igem.org/Team_List?year=all [Accessed 31 August 2022].

7 iGEM Foundation (2021b). iGEM Design League. Available at: https://old.igem.org/Designleague/jamboree#meet-teams [Accessed 30 August 2022].

8 iGEM Foundation (2009). Team List For iGEM 2009 Championship. Available at: https://old.igem.org/Team_List?year=2009&name= Championship&division=igem [Accessed 27 August 2022].

9 iGEM Foundation (2022). Team List For All Years. Team List For All Years. Available at: https://old.igem.org/Team_List?year=all [Accessed 31 August 2022].

10 iGEM Foundation (2014). 2014 Jamboree Awards. Available at: https://old.igem.org/2014_Results [Accessed 20 November 2022].

11 iGEM Foundation (2017). 2017 Competition Results. Available at: https://2017.igem.org/Competition/Results [Accessed 20 November 2022].

12 iGEM Foundation (2018). 2018 Competition Results. Available at: https://2018.igem.org/Competition/Results [Accessed 20 November 2022].

13 iGEM Foundation (2019). 2019 Competition Results. Available at: https://2019.igem.org/Competition/Results [Accessed 20 November 2022].

14 iGEM Foundation (2021). iGEM Grand Jamboree. Available at: https://jamboree.igem.org/2021/results/tracks [Accessed 20 November 2022].

15 UdelaR (2020). Programa de Bioingeniería Celular y Molecular. Programa de Bioingeniería Celular y Molecular. Available at: https://www.colibri.udelar.edu.uy/jspui/handle/20.500.12008/27126 [Accessed 31 August 2022].

16 SMS (2019). SMS: Simposio de Microbiología Sintética. Available at: https://vanesha07.wixsite.com/symmicrobio [Accessed 31 August 2022].

17 Bioinformáticos (2012). Curso de Introducción a la Biología Sintética. Bioinformáticos Curso de Introducción a la Biología Sintética. Available at: http://www.bioinformaticos.com.ar/curso-de-introduccion-a-la-biologia-sintetica/[Accessed 31 August 2022].

18 iGEM Foundation (2012a). iGEM BsAs. Team:Buenos Aires. Available at: https://2012.igem.org/Team:Buenos_Aires/Team/BsAs [Accessed 31 August 2022].

19 iGEM Foundation (2013). Team Buenos Aires 2013. Team:Buenos Aires. Available at: https://2013.igem.org/Team:Buenos_Aires/_summary [Accessed 31 August 2022].

20 iGEM Foundation (2014). Mercury Bacter. Team:UFAM Brazil/Argentina. Available at: https://2014.igem.org/Team:UFAM_Brazil/Argentina [Accessed 30 August 2022].

21 iGEM Foundation (2021b). iGEM Design League. Available at: https://old.igem.org/Designleague/jamboree#meet-teams [Accessed 30 August 2022].

22 Cabezas, C. (2013). Charla de Ron Weiss “Biología Sintética: desde partes a módulos a aplicaciones terapéuticas”/UAI - Chile. Available at: https://www.archdaily.co/co/02-231481/charla-de-ron-weiss-biologia-sintetica-desde-partes-a-modulos-a-aplicaciones-terapeuticas-uai-chile [Accessed 11 September 2022].

23 UC (2022a). Biología Sintética (2do semestre 2022). Available at: https://postgrado.bio.uc.cl/educacion/cursos/bio4026-biologia-sintetica/[Accessed 11 September 2022].

24 UC (2022b). Biología Sintéticas: Maquinas Biológicas. Available at: http://pregrado.bio.uc.cl/cursos/biologia-sinteticas-maquinas-biologicas/[Accessed 11 September 2022].

25 iGEM Foundation (2016). iGEM FDR. Team:ColegioFDR Peru. Available at: https://2016.igem.org/Team:ColegioFDR_Peru [Accessed 31 August 2022].

26 iGEM Foundation (2020). Crio Prot. Team:UPCH Peru. Available at: https://2020.igem.org/Team:UPCH_Peru [Accessed 31 August 2022].

27 Rocha, P. (2020). Generalidades sobre la Biología Sintética.

28 Proaño (2014). Charla de Biología Sintética y iGEM.

29 iGEM Foundation (2018). C-lastin. Team:Ecuador. Available at: https://2018.igem.org/Team:Ecuador [Accessed 31 August 2022].

30 iGEM Foundation (2021a). Agrobactory 593 Awards. Team:Ecuador awards. Available at: https://2021.igem.org/Team:Ecuador/awards [Accessed 31 August 2022].

31
https://leagues.igem.org/design/2022/seasonfinale

32
http://www.investigacionsalud.gob.ec/inspi-realiza-seminario-sobre-tecnicas-de-biologia-sintetica-aplicadas-en-el-diagnostico-rapido-de-zika/

33
http://www.quitoinforma.gob.ec/2022/07/12/participe-del-cafe-cientifico-y-conozca-mas-sobre-la-biotecnologia-en-ecuador/

34
https://www.cebioec.com/event-details/biologia-sintetica-metodos-experimentales-para-la-construccion-de-circuitos-biologicos-in-silico

35 DC UBA (2018). Se realizó TECNOx: una competencia científica innovadora. Available at: https://www.dc.uba.ar/se-realizo-tecnox-una-competencia-cientifica-innovadora/[Accessed 10 October 2022].

36 OECD (2009). The bioeconomy to 2030: designing a policy agenda. OECD doi:10.1787/9789264056886-en.

37 CNI (2013). BIOECONOMY An Agenda for Brazil. Available at: http://arquivos.portaldaindustria.com.br/app/conteudo_24/2013/10/18/411/20131018135824537392u.pdf [Accessed August 31, 2022].

38 Silva, M. F., Pereira, F., and Martins, J. V. B. (2018). A bioeconomia Brasileira em Números 2018. Available at: https://web.bndes.gov.br/bib/jspui/bitstream/1408/15383/1/BS47__Bioeconomia__FECHADO.pdf [Accessed 11 September 2022].

39 The University of Warwick School of Life Sciences (2015). Warwick extends synthetic biology collaborations in Brazil. Available at: https://warwick.ac.uk/fac/sci/lifesci/news/?newsItem=094d43454ef9384d014f082cb05976d3 [Accessed 31 August 2022].

40 Gobierno de Argentina (2021). Bioeconomía Argentina. Available at: https://www.argentina.gob.ar/agricultura/bioeconomia [Accessed 31 August 2022].

41 AST (2010). ARGENTINA. Available at: https://ebrary.ifpri.org/digital/api/collection/p15738coll2/id/999/download [Accessed 11 September 2022].

42 Haar, J. (2017). Ecosystems of Innovation: The Case of Biotechnology in Argentina. Available at: https://www.wilsoncenter.org/sites/default/files/media/documents/misc/biotechnology_in_arg_haar.pdf [Accessed 11 September 2022].

43 Anlló, G., and Carri, R. (2016). Biotecnología argentina al año 2030: llave estratégica para un modelo dedesarrollo tecno-productivo. Available at: https://www.argentina.gob.ar/sites/default/files/est_bio_biotecnologia-argentina-al-2030-sintesis.pdf [Accessed 11 September 2022].

44 Verre, V., Milesi, D., and Petelski, N. (2022). Academic, Сommercialization and Societal Effects of Joint Research. FORESIGHT AND STI GOVERNANC 16.

45 UNESCO (2022). Gasto en investigación y desarrollo (% del PIB) - Chile. World Bank.

46 FONDAP (2015). Historia Fondap Fondo de Financiamiento de Centros de Investigación en Áreas Prioritarias. Available at: https://www.conicyt.cl/fondap/sobre-fondap/historia/[Accessed 11 September 2022].

47 FONDAP (2021). Centros FONDAP. Available at: https://www.anid.cl/centros-e-investigacion-asociativa/centros-fondap/[Accessed 11 September 2022].

48 NMBSSV (2014). Millennium Nucleus Center for Plant Systems and Synthetic Biology. Millenium Nucleus Plants Systems and Synthetic Biology. Available at: http://virtualplant.bio.puc.cl/cgi-bin/NMBSSV/indexENG.cgi [Accessed 10 September 2022].

49 PEDECIBA (2022). Programa de Desarrollo de las Ciencias Básicas PEDECIBA. Available at: https://www.pedeciba.edu.uy/en/institucional/ [Accessed 31 August 2022].

50 CNRS (2019). La investigación en Uruguay. Available at: https://www.cnrsrio.org/es/lorganisation-de-la-recherche-en-amerique-du-sud/recherche-en-uruguay/ [Accessed 31 August 2022].

51 Ehrlich, R., Lorenzo, F., Kreimerman, R., Venegas, J., Aguerre, T., and Frugoni, G. (2012). Ciencia, Tecnología e Innovación en Uruguay en los últimos años. Available at: https://www.gub.uy/ministerio-educacion-cultura/sites/ministerio-educacion-cultura/files/documentos/publicaciones/ciencia-tecnologia-innovacion-uruguay-ultimos-anios.pdf [Accessed 11 September 2022].

52 ANII (2021). Reprogramación bacteriana a través de la ingeniería de sistemas de señalización. Reprogramación bacteriana a través de la ingeniería de sistemas de señalización. Available at: https://www.anii.org.uy/proyectos/FCE_1_2021_1_166888/reprogramacion-bacteriana-a-traves-de-la-ingenieria-de-sistemas-de-senalizacion/ [Accessed 27 June 2022].

53 UNESCO (2021). Gasto en investigación y desarrollo (% del PIB) - Uruguay. World Bank.

54 Oliveros-Grijalva, M. (2003). Fundación Consorcio Ecuatoriano para el Desarrollo de Internet Avanzado. Available at: https://www.cedia.edu.ec/assets/docs/10-01-03.pdf [Accessed 11 September 2022].

55
https://www.espe.edu.ec/ingenieria-en-biotecnologia/

56
https://www.udla.edu.ec/carreras/programas-academicos/pregrados/facultad-de-ingenieria-y-ciencias-aplicadas/ingeniero-en-biotecnologia/

57
https://www.ikiam.edu.ec/index.php/pregado/facultad_ciencias_vida/biotecnologia/

58
https://biotecnologia.utn.edu.ec/

59
https://www.usfq.edu.ec/es/carreras-de-pregrado/biotecnologia

60
https://puceapex.puce.edu.ec/pucevirtual/maestria-online-en-biologia-computacional/

61 Gallardo-Sinchiguano, L. N. (2022). Inversión en I + D + i en Sudamérica: Un análisis multivariante. Available at: https://repositorio.uta.edu.ec/bitstream/123456789/34446/1/T5247e.pdf [Accessed 11 September 2022].

62 CONICET (2021). Las startups que posicionan a Argentina como líder en biotecnología se lucieron en la BioArgentina 2021. Available at: https://www.conicet.gov.ar/las-startups-que-posicionan-a-argentina-como-lider-en-biotecnologia-se-lucieron-en-la-bioargentina-2021/ [Accessed 31 August 2022].

63 INMET (2011). INMET | Ingeniería Metabólica. Available at: https://inmet.com.ar/ [Accessed 8 July 2022].

64 Syocin (2019). Syocin | Biotech. Available at: https://syocin.com/ [Accessed 8 July 2022].

65 Andes (2022). Our Platform Andes. Available at: https://www.andes.bio/platform [Accessed 11 September 2022].

66 Neumann, S. (2021). Andes, la biotech con ADN chileno que quiere impactar en el cambio climático, apalanca US$ 15 millones. Startups Latam.

67 SilicoChem (2019). SilicoChem: Let’s make good chemistry. Available at: https://www.silico-chem.com/ [Accessed 8 July 2022].

68 GRIDX (2022). GRIDX. Available at: https://www.gridexponential.com/ [Accessed 14 December 2022].

69 The Ganesha Lab (2022). The Ganesha Lab. Available at: https://theganeshalab.com/ [Accessed 14 December 2022].

70 Dorocki, S., and Wójtowicz, M. (2014). Regional differences in the development of the biotechnology industry in Latin America, with particular emphasis on Brazil and Mexico., eds. M. Wójtowicz and A. Winiarczyk-Raźniak Wydawnictwo Naukowe UP.

71 Sanchez-Barba, G. (2014). WE ARE BIOHACKERS: EXPLORING THE COLLECTIVE IDENTITY OF THE DIYBIO MOVEMENT. Available at: https://www.researchgate.net/profile/Gabriela-Sanchez-5/publication/284727537_We_are_Biohackers_Exploring_the_Collective_Identity_of_the_DIYbio_Movement/links/565845c808aefe619b20e07f/We-are-Biohackers-Exploring-the-Collective-Identity-of-the-DIYbio-Movement.pdf [Accessed 17 August 2022].

72 Lemarchand, G. A. (2010). Science, Technology and Innovation Systems in Latin America and the Caribbean. Available at: https://unesdoc.unesco.org/ark:/48223/pf0000189823 [Accessed 11 September 2022].

73 Kera, D. (2015). Open Science, open issues. Available at: https://wlu18www30.webland.ch/wiki/images/6/6c/Open_Science_2015_Kera_GeekDiplomacy.pdf [Accessed 11 September 2022].

74 Dolabella, M., and Durán-Lima, J. E. (2021). Integrating Latin America and the Caribbean. Available at: https://repositorio.cepal.org/bitstream/handle/11362/46649/1/S2000597_en.pdf [Accessed 11 September 2022].

75 Fuller, V. (2015). Why is branded tech so expensive in Argentina? Available at: https://www.idgconnect.com/article/3581823/why-is-branded-tech-so-expensive-in-argentina.html [Accessed 11 September 2022].

76 Hilbert, M. R. (2003). Latin America On Its Path Into The Digital Age: Where Are We? Available at: https://repositorio.cepal.org/bitstream/handle/11362/4484/S01060494_en.pdf [Accessed 11 September 2022].

77 OECD/WBG/CIAT/IDB (2021). VAT Digital Toolkit for Latin America and the Caribbean. Available at: https://www.oecd.org/tax/consumption/vat-digital-toolkit-for-latin-america-and-the-caribbean.pdf [Accessed 11 September 2022].

78 Ramírez, C. (2022). Understanding Complex Regulatory Landscapes for Consumer Electricals in Latin America. Available at: https://www.sgs.com/en/news/2022/03/understanding-complex-regulatory-landscapes-for-consumer-electricals-in-latin-america [Accessed 11 September 2022].

79 Selman, C., Caicedo, C., Sevilla-Macip, J., and Retamales-Burdford, V. (2022). Supply chains disruptions in Latin America. Available at: https://ihsmarkit.com/research-analysis/supply-chains-disruptions-in-latin-america.html [Accessed 11 September 2022].

80 Medrano-Villalobos, A. J., and Chourio-Acevedo, L. M. (2017). HARDWARE LIBRE Y ABIERTO,MODELOS DE NEGOCIOS PARA AMÉRICALATINA EN EL CARIBE. Available at: https://www.lajbm.com.br/index.php/journal/article/download/407/191 [Accessed 11 September 2022].

81 Metamaquina (2021). Metamaquina GitHub. Available at: https://github.com/Metamaquina [Accessed 1 September 2022].

82 Herrera‐Juarez, P. and Juárez, B. (2013). Fabrication Laboratories: Problems and possibilities of implementation in Latin America. Available at: https://citeseerx.ist.psu.edu/viewdoc/download?doi=10.1.1.635.3485&rep=rep1&type=pdf [Accessed September 18, 2022].

83 Abeliuk, E. (2021). Growing Synbio Talent in Latin America with iGEM Design League. Teselagen Biotechnology. Available at: https://teselagen.com/blog/growing-synbio-talent-in-latin-america-with-igem/ [Accessed 7 September 2022].

84 Arancio, J. (2018). GOSH Roadmap: democratizing technology, from a Latin American perspective. Steps Center Pathways to Sustainability. Available at: https://steps-centre.org/blog/gosh-roadmap-democratizing-technology-latin-american-perspective/ [Accessed 7 September 2022].

85 Georgia Tech (2022). Biotechnology for public health and economic development in the Americas. Available at: http://jordan.biology.gatech.edu/page/BioLatinoAmerica.pdf [Accessed 7 September 2022].

86 Heng, A., Heermann, J., Samaranayake, C., and Sath, L. (2010). Technology Trends in Latin America and Their Social and Economic Impact. Technology Trends in Latin America. Available at: https://cs.stanford.edu/people/eroberts/cs181/projects/2010-11/TechnologyTrendsLatinAmerica/index-2.html [Accessed 7 September 2022].

87 de Lange, O., Dunn, K., and Peek, N. (2022). “Short on time and big on ideas”: Perspectives from Lab Members on DIYBio Work in Community Biolabs. Available at: https://arxiv.org/pdf/2205.00079.pdf [Accessed 7 September 2022].

88 Medrano-Villalobos, A. J., and Chourio-Acevedo, L. M. (2017). HARDWARE LIBRE Y ABIERTO,MODELOS DE NEGOCIOS PARA AMÉRICALATINA EN EL CARIBE. Available at: https://www.lajbm.com.br/index.php/journal/article/download/407/191 [Accessed 11 September 2022].

89 Flores, L. (2018). DIY BIO (Hazlo Tú Mismo—Bio). Available at: https://utec.edu.pe/blog-de-carreras/bioingenieria/diy-bio-hazlo-tu-mismo-bio [Accessed 7 September 2022].

90 Innovación Ciudadana (2022). Civic Innovation. Available at: https://www.innovacionciudadana.org/en/ [Accessed 7 September 2022].

91 Lab de tecnología libre (2022). Lab de tecnología libre. Available at: https://federicilab.org/about/ [Accessed 1 September 2022].

92 RedFungi (2022). RedFungi. Available at: https://redfungiblog.wordpress.com/que_hacemos/ [Accessed 1 September 2022].

93 Cerdas, R., Céspedes, C., and Cortés, M. (2017). Los laboratorios de innovación desde una perspectiva de Gobierno Abierto. Available at: https://transparencia.ucr.ac.cr/medios/documentos/2017/informe_laboratorios_de_innovacion.pdf [Accessed 9 September 2022].

94 Sperling, D. M., Herrera, P. C., and Scheeren, R. (2015). International Conference on Computer-Aided Architectural Design Futures. Available at: https://repositorioacademico.upc.edu.pe/bitstream/handle/10757/614909/chapter-3-662-47386-3_22.pdf?sequence=1&isAllowed=y [Accessed 9 September 2022].

95 Sperling, D. M., Herrera, P. C., and Scheeren, R. (2015). International Conference on Computer-Aided Architectural Design Futures. Available at: https://repositorioacademico.upc.edu.pe/bitstream/handle/10757/614909/chapter-3-662-47386-3_22.pdf?sequence=1&isAllowed=y [Accessed 9 September 2022].

96 Fab Lab Peru (2019). Fab Lab Perú: Una década de Fab Labs en el Perú. Available at: https://www.fab.pe/homepages/nosotros/ [Accessed 1 September 2022].

97 UTEC (2018). Bioacademy 2017: Biología Sintética al Máximo en UTEC. Available at: https://utec.edu.pe/blog-de-carreras/bioingenieria/bioacademy-2017-biologia-sintetica-al-maximo-en-utec [Accessed 1 September 2022].

98 Taday, S. (2022). Biotec Latina. Available at: https://www.facebook.com/BiotecLatina/ [Accessed 1 September 2022].

99 LABVA (2022). Somos LABVA. Available at: https://www.labva.org/somoslabva/ [Accessed 1 September 2022].

100 Etimo (2020). Etimo Inicio. Available at: https://etimobiomateria.wixsite.com/etimo [Accessed 1 September 2022].

101 UBA (2021). Biodesign Challenge. Available at: https://www.biodesignchallenge.org/uba-2021 [Accessed 31 August 2022].

102 102 Pérez-Sedeño, E. and Gómez, A. (2008). Equality and Equity in LatinAmerican Science and Technology. Available at: https://arbor.revistas.csic.es/index.php/arbor/article/download/223/224/224 [Accessed September 18, 2022].

103 MarketDataForecast (2022). Latin America Next-Generation Sequencing Market Research Report - By Product, By Technology, by Application, By Country (Brazil, Argentina, Chile & Rest of Latin America) - Industry Analysis on Size, Share, Trends, COVID-19 Impact & Growth Forecast (2022–2027). Available at: https://www.marketdataforecast.com/market-reports/latin-america-next-generation-sequencing-market [Accessed 14 December 2022].

104 MarketDataForecast (2022). Latin America Next-Generation Sequencing Market Research Report - By Product, By Technology, by Application, By Country (Brazil, Argentina, Chile & Rest of Latin America) - Industry Analysis on Size, Share, Trends, COVID-19 Impact & Growth Forecast (2022–2027). Available at: https://www.marketdataforecast.com/market-reports/latin-america-next-generation-sequencing-market [Accessed 14 December 2022].

105 Globocan (2020). Latin America and the Caribbean Hub. World Health Organization.

106 Google Cloud (2022). DNA Do Brasil Case Study. Available at: https://cloud.google.com/customers/dna-do-brasil [Accessed 14 December 2022].

107 AST (2010). ARGENTINA. Available at: https://ebrary.ifpri.org/digital/api/collection/p15738coll2/id/999/download [Accessed 11 September 2022].

108 CONICET (2021). Las startups que posicionan a Argentina como líder en biotecnología se lucieron en la BioArgentina 2021. Available at: https://www.conicet.gov.ar/las-startups-que-posicionan-a-argentina-como-lider-en-biotecnologia-se-lucieron-en-la-bioargentina-2021/ [Accessed 31 August 2022].

109 ISAAA (2019). Global Status of Commercialized Biotech/Gm Crops in 2019. Philippines: ISAAA.

110 Mittermeier, R. A., and Mittermeier, C. G. (2005). Megadiversity: Earth’s Biologically Wealthiest Nations. 1st ed. México, D.F.]: CEMEX.

111 Balakrishnan, R., Lizarazo Ruiz, S., Santoro, M., Toscani, F., and Vargas, M. (2021). Commodity cycles, inequality, and poverty in Latin America. Available at: https://www.elibrary.imf.org/view/journals/087/2021/009/article-A001-en.xml [Accessed 23 September 2022].

112 Wolrd Bank (2021). GDP per Country. Available at: https://data.worldbank.org/indicator/NY.GDP.MKTP.CD?locations=BR [Accessed 16 September 2022].
